# The Prognostic Role of Para-Aortic Lymph Nodes in Patients with Colorectal Cancer: Is It Regional or Distant Disease?

**DOI:** 10.1371/journal.pone.0130345

**Published:** 2015-06-26

**Authors:** Hsueh-Ju Lu, Jen-Kou Lin, Wei-Shone Chen, Jeng-Kai Jiang, Shung-Haur Yang, Yuan-Tzu Lan, Chun-Chi Lin, Chien-An Liu, Hao-Wei Teng

**Affiliations:** 1 Division of Hematology and Oncology, Show Chwan Memorial Hospital, Changhua, Taiwan; 2 Division of Colon and Rectum Surgery, Department of Surgery, Taipei Veterans General Hospital, Taipei, Taiwan; 3 Department of Radiology, Taipei Veterans General Hospital, Taipei, Taiwan; 4 Division of Hematology and Oncology, Department of Medicine, Taipei Veterans General Hospital, Taipei, Taiwan; 5 Institute of Clinical Medicine, National Yang-Ming University, Taipei, Taiwan; 6 School of Medicine, National Yang-Ming University, Taipei, Taiwan; 7 Program in Molecular Medicine, School of Life Sciences, National Yang-Ming University, Taipei, Taiwan; Mie University, JAPAN

## Abstract

**Introduction:**

Visible para-aortic lymph nodes of ≥2 mm in size are common metastatic patterns of colorectal cancer (CRC) seen on imaging. Their prognostic value, however, remains inconclusive. We aimed to assess the prognostic role of visible para-aortic lymph nodes (PALNs).

**Methods:**

Patients with confirmed pathologic diagnosis of CRC were enrolled. Correlations among clinicopathologic variables were analyzed using the χ^2^ test. The Cox proportional hazards model was applied for univariate and multivariate analyses. Survival was estimated using the Kaplan-Meier method and log-rank test. A prognostic model for visible PALNs in CRC patients was established.

**Results:**

In total, 4527 newly diagnosed CRC patients were enrolled. Patients with visible PALNs had inferior overall survival compared to those without visible PALNs (5-year overall survival, 67% vs. 76%, *P* = 0.015). Lymphovascular invasion (LVI) (hazard ratio = 1.865, *P* = 0.015); nodal disease (pN+) status (hazard ratio = 2.099, *P* = 0.006); elevated preoperative serum carcinoembryonic antigen (CEA) levels (hazard ratio = 2.263, *P* < 0.001); and visible PALNs ≥10 mm (hazard ratio = 1.638, *P* = 0.031) were independent prognostic factors for patients with visible PALNs. If each prognostic factor scored one point, 5-year overall survival of lower- (prognostic score 0–1), intermediate- (prognostic score 2), and high- (prognostic score 3–4) risk groups were, 78%. 54%, and 25% respectively (*P* < 0.001).

**Conclusions:**

The prognostic model, which included LVI, pN+ status, preoperative serum CEA level, and the size of visible PALNs, could effectively distinguish the outcome of patients with visible PALNs.

## Introduction

Globally, colorectal cancer (CRC) is the fourth and third most common cancer in men and women respectively [1]. The survival and treatment strategies for patients with CRC correlate with disease-stage status. Adequate treatments would lead to long-term survival, even for advanced-stage patients [2, 3].

With the improvement of imaging modalities, such as computed tomography (CT) and magnetic resonance imaging (MRI), enlarged para-aortic lymph nodes (PALNs) (so-called visible PALNs) are a more commonly observed metastatic pattern in CRC [4]. However, according to the American Joint Committee on Cancer (AJCC) staging system, visible PALN metastases are categorized as clinical stage M1 because they are considered to be non-regional lymph nodes [5]. There have been no original reports addressing the impact of visible PALNs on the clinical behavior of CRC and the survival of patients. Moreover, although some articles have mentioned the poor prognostic value of visible PALNs in recurrence [6], the prognostic role of visible PALNs at initial diagnosis by modern imaging studies remains unclear.

In contrast, extensive surgical dissection and radiation therapy reportedly increases the survival of selected patients with visible PALN metastases. These patients were treated following curative resection or loco-regional recurrence [7–9]. However, dissection of PALNs is difficult, and the incidence of postoperative complications after extensive lymph node dissection is relatively high [4, 10, 11]. Therefore, it remains unclear to clinicians whether visible PALNs in CRC patients represent regional or distant disease; and consequently, whether aggressive treatments such as surgical LN dissection or chemoradiotherapy should be arranged for patients with visible PALN enlargement on initial imaging diagnosis. Thus, the association between visible PALNs and clinic-pathological parameters requires clarification. There is currently, not enough data to stratify patients for aggressive treatment.

In this study, we aimed to assess the prognostic role of visible PALNs in patients with CRC, and attempted to establish a prognostic model for visible PALNs. Although this model could not predict pathologic metastasis of PALNs, it could help us to predict outcomes for CRC patients in combination with the observation of visible PALNs on imaging studies. With this model, we can modify our clinical practice and select some patients with visible PALNs for management.

## Materials and Methods

### Study design, setting, and patient selection

The study was a single institute, retrospective, cohort study. In this study, all data were collected according to routine clinical care in our hospital. There was no direct contact with patients for any data collection and analysis; as such, the need for written consent from study subjects was waived by the institutional review board. The study was reviewed and approved by the Institutional Review Board of Taipei Veterans General Hospital (No. 2012-11-004BC).

Between January 2001 and December 2011, patients with clinically suspected CRC were selected for advanced survey. Patients with pathologically confirmed CRC at Taipei Veterans General Hospital were enrolled in our database. Patients with no pathologically proven CRC, carcinoma in situ, malignancies other than adenocarcinoma, and secondary primary malignancies were excluded from our database. In order to describe the prognostic role of visible PALNs in CRC, we selected patients without distal metastases and divided them into patient groups with, or without, visible PALNs. Patients with visible PALNs, but no distal metastases (such as lung and liver metastases), were categorized in the visible PALNs group. The remaining patients were categorized as without visible PALNs, and were staged according to the AJCC staging system, 6th edition, and National Comprehensive Cancer Network guidelines [5].

### Definition of visible para-aortic lymph nodes

All patients received radiologic (such as CT and MRI) examinations at initial diagnosis. The identification of visible PALNs was retrospectively reviewed from imaging records. All images were read independently by two experienced abdominal radiologists, to determine the short axis diameter of lymph nodes and reach a consensus via discussion. A third radiologist evaluated the lesions if there were inconsistencies in visible PALN identification. Visible PALNs were defined as lymph nodes surrounding the abdominal aorta and inferior vena cava, which were located in the area from the uppermost part of the origin of the celiac trunk to the lower margin of the aortic bifurcation [12]. Visible PALNs measured more than 2 mm in the short axis and were detectable on radiologic examinations.

### Imaging Technique

CT images were obtained using a standardized acquisition protocol covering the abdomen and pelvis on multiple row-detector CT systems in all selected cases. Reconstructions were performed in 5.0 mm slice thickness. For lymph nodes measuring less than 5.0mm on reconstructed CT images, additional thin-section CT with less than 1.5mm slice thickness was obtained from the data stored in the server of the PACS system and reviewed at the site of target lesions. MRI imaging was performed on 1.5T MR unit using a pelvic phased-array body coil at a slice thickness of 5 mm and 1mm gap. All images were read independently by experienced abdominal radiologists who determined short axis diameter of lymph nodes and reach the consensus via discussion.

### Data collection

Basic clinicopathologic parameters were recorded including: age, gender, stage, tumor location, pathologic features (e.g., histological type, lymphovascular invasion, perineural invasion, and grade), and preoperative serum carcinoembryonic antigen (CEA) levels. The descriptions of visible PALNs (e.g., the largest short axis diameter, number, side) were also recorded according to the image reports. The best supportive care was defined as patients who receive treatment administered with the intent to maximize quality of life without a specific antineoplastic regimen. This included antibiotics, analgesics, antiemetics, thoracentesis, pleurodesis, blood transfusions, nutritional support, and focal external-beam radiation for control of pain, cough, dyspnea, or hemoptysis [13]. Overall survival (OS) was calculated from the date of disease diagnosis to the date of death or the date on which the patient was last evaluated. The final follow-up date was December 31, 2012.

### Statistical analysis

The correlations among clinicopathologic variables were analyzed using the χ^2^ test or Fisher exact test. The Cox proportional hazards model was applied for univariate and multivariate analyses. Survival was estimated using the Kaplan-Meier method, and the log-rank test was used for the comparison of survival curves. Variables with *P* values <0.05 in univariate analyses were entered into multivariate analysis models. A two-sided *P* value <0.05 was regarded as statistically significant. SPSS statistical software (version 18.0, SPSS Inc., Chicago, IL, USA) was used for all statistical analyses.

## Results

### Baseline characteristics of patients with or without visible PALNs

**Fig 1:**
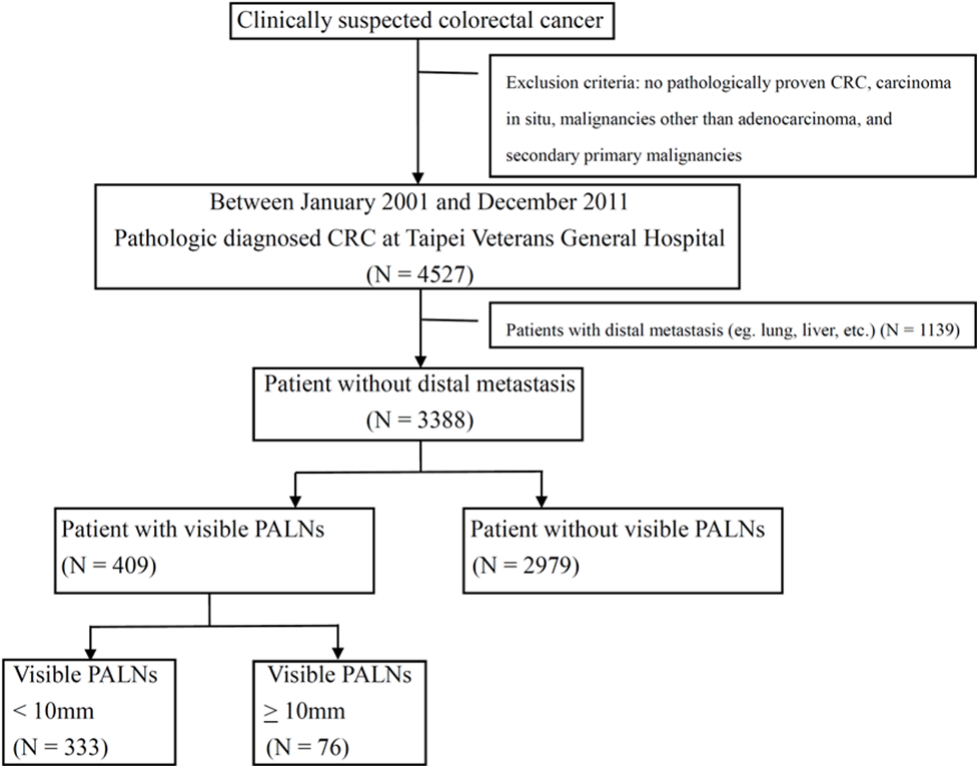
Flow chart of patient enrollment and exclusion. There were 4527 newly diagnosed CRC patients in our institution, including 1139 stage IV CRC with distal metastases. Only 3388 patients without distal metastases were selected and divided into the groups with or without visible PALNs. Four hundred and nine patients were identified as having visible PALNs without distal metastases.

**Fig 2:**
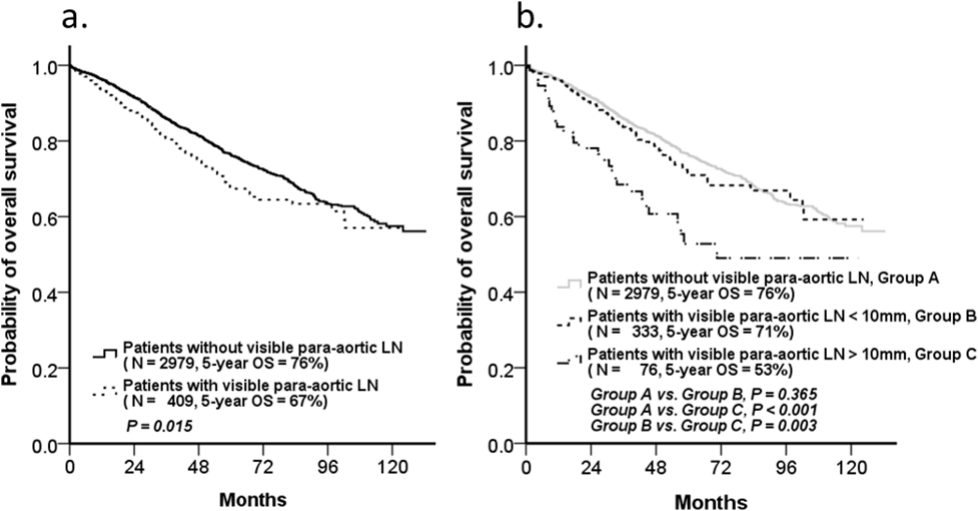
Overall survival (OS) of CRC patients with or without visible PALNs. (a) Patients with visible PALNs showed significantly poorer overall survival than those without visible PALNs (5-year OS, 67% vs. 76%*P* = 0.015). (b) But the survivals between patients with visible PALNs < 10 mm and those without visible PALNs were the same (5-year OS, 71% vs. 76%, *P* = 0.365).

Between January 1, 2001 and December 31, 2011, there were 4527 newly diagnosed CRC patients in our institution, including 1139 stage IV CRC with distal metastases. The presence of distal metastases is significantly associated with poor patient prognoses for CRC; therefore, only 3388 patients without distal metastases were selected and divided into the groups with or without visible PALNs. Four hundred and nine patients were identified as having visible PALNs without distal metastases. There were 106 patients with visible PALNs who died during follow up. The flow chart of patient enrollment and exclusion is shown in Fig 1. The basic characteristics of patients with and without visible PALNs are presented in Table 1 and S1 Table. Patients with visible PALNs were significantly younger (*P* = 0.014), predominantly male (*P* = 0.014), and had more lymphovascular invasion (*P* < 0.001), advanced pathologic staging (pT, *P* < 0.001; pN, *P* < 0.001), and elevated preoperative serum CEA levels (*P* = 0.021) (Table 1). The OS of patients with visible PALNs compared to patients without visible PALNs was significantly shorter (5-year OS, 67% vs. 76%, *P* = 0.015) (Fig 2A).

**Table 1:** Characteristics of patients with or without visible PALNs^a^.

Characteristics (N = 3388)			Patients without visible PALNs	Patients with visible PALNs	*P* value
			N = 2979 (%)	N = 409 (%)	
Age (years)	< 65		1075 (36.1)	171 (41.8)	0.014
	≥ 65		1904 (63.9)	238 (58.2)	
Gender	Male		1878 (63.0)	281 (68.7)	0.014
	Female		1101 (37.0)	128 (31.3)	
Tumor Location	Colon		2192 (73.6)	301 (73.6)	0.525
	Rectum		787 (26.4)	108 (26.4)	
Histological type	Adenocarcinoma		2817 (94.6)	286 (94.4)	0.704
	Mucinous adenocarcinoma		115 (3.9)	14 (3.4)	
	Signet ring cell adenocarcinoma		40 (1.3)	7 (1.7)	
	Carcinoma, NOS		7 (0.2)	2 (0.5)	
Primary tumor	Lymphovascular invasion	Negative	2645 (88.8)	338 (82.6)	<0.001
		Positive	334 (11.2)	71 (17.4)	
	Perineural invasion	Negative	2886 (96.9)	393 (96.1)	0.236
		Positive	93 (3.1)	16 (3.9)	
	Grade^b^	Lower	2734 (91.8)	369 (90.2)	0.166
		High	245 (8.2)	40 (9.8)	
Pathologic staging	pT	1	435(14.6)	28(6.8)	<0.001
		2	462(15.5)	50(12.2)	
		3	1905(63.9)	288(70.4)	
		4	177(5.9)	43(10.5)	
	pN	N0	1974 (66.3)	220 (53.8)	<0.001
		N+	1005 (33.7)	189 (46.2)	
Preoperative serum CEA level (ng/mL)	<10		2424 (85.3)	322 (81.1)	0.021
	≥10		419 (14.7)	75 (18.9)	
	Data available		2843	397	
5-year overall survival			76%	67%	0.015

^a^All patients were diagnosed as having no distant metastases

^b^Lower grade represents well or moderately differentiated histology and high grade represents poorly differentiated histology or mucinous carcinoma.

*PALNs*, *para-aortic lymph nodes; CRC*, *colorectal cancer; NOS*, *not otherwise specified; CEA*, *carcinoembryonic antigen*

### The impact of visible PALNs

Univariate and multivariate Cox proportional hazards regression models were analyzed to identify the impact of visible PALNs. We classified patients >65 years of age as elderly. The management of elderly CRC patients is an important issue because of more comorbidities and poor performance status [14]; the dosage of treatment for these patients should be modified [15, 16]. Although many factors influence the CEA levels [17], rare conditions result in elevated CEA levels exceeding 10ng/mL due to non-malignancy disease, including inflammation [18]. Therefore, the cut-off CEA level was set at 10 ng/mL. This analysis demonstrated that the presence of visible PALNs was only significant in univariate (*P* = 0.015) analysis, but was not an independent prognostic factor in multivariate Cox regression analysis for OS (hazard ratio [HR] = 1.198, 95% confidence interval [CI] = 0.970–1.480, *P* = 0.094) (Table 2).

**Table 2:** Univariate and multivariate Cox regression analysis of prognostic factors for overall survival in patients without distal metastases.

Characteristics (N = 3388)		Univariate	Multivariate	
		*P* value	*P* value	HR (95% CI)
Age (years) ≥65		<0.001	<0.001	2.283 (1.912–2.725)
Male		<0.001	<0.001	1.407 (1.196–1.656)
Rectal cancer		0.044	0.001	1.318 (1.128–1.541)
Primary tumor	Lymphovascular invasion	<0.001	0.006	1.334 (1.084–1.641)
	Perineural invasion	<0.001	0.006	1.620 (1.149–2.286)
	High grade^a^	<0.001	0.005	1.368 (1.100–1.700)
Pathologic staging	pT >2	<0.001	0.011	1.292 (1.062–1.572)
	pN+	<0.001	<0.001	1.661 (1.417–1.946)
Preoperative serum CEA (ng/mL) level ≥10		<0.001	<0.001	1.568 (1.316–1.868)
Visible PALNs (≥2 mm)		0.015	0.094	1.198 (0.970–1.480)

^a^High grade represents poorly differentiated histology or mucinous carcinoma.

*PALNs*, *para-aortic lymph nodes; CRC*, *colorectal cancer; HR*, *hazard ratio; CI*, *confidence interval; CEA*, *carcinoembryonic antigen*

### Analysis of prognostic factors in patients with visible PALNs

Because the presence of visible PALNs was not an independent prognostic factor for OS and the survivals between patients with visible PALNs < 10 mm and those without visible PALNs were the same (5-year OS, 71% vs. 76%, *P* = 0.365) (Fig 2B), it was important to identify the true poor prognostic factors in patients with visible PALNs to provide the most appropriate treatment. In the past, visible PALNs measuring ≥10 mm in the short axis on radiologic examinations were considered a significant metastatic finding [19]. Therefore we compared the characteristics of patients with, or without, visible PALNs measuring >10 mm in the short axis. Our analysis showed that patients with larger PALNs had more lymphovascular invasion (*P* = 0.004), higher grade disease (*P* = 0.019), and more regional lymph node metastasis (*P* = 0.001). There were no significant differences between the two patient groups in the percentage of patients who received neoadjuvant chemotherapy (*P* = 0.123), and neoadjuvant concurrent chemoradiotherapy (CCRT) (*P* = 0.218). Although a significantly higher proportion of patients in the visible PALNs >10 mm group received postoperative chemotherapy (*P* < 0.001), the role of postoperative chemotherapy (either adjuvant chemotherapy or palliative chemotherapy) was still unclear because the prognostic role of visible PALNs was not evident (Table 3).

**Table 3:** Characteristics of patients with visible PALNs.

Characteristics (N = 409)			Diameter of visible PALNs <10 mm	Diameter of visible PALNs ≥10 mm	*P* value
			N = 333 (%)	N = 76 (%)	
Age (years)	<65		141 (42.3)	30 (39.5)	0.373
	≥65		192 (57.7)	46 (60.5)	
Gender	Male		233 (70.0)	48 (63.2)	0.154
	Female		100 (30.0)	28 (36.8)	
Tumor Location	Colon		241 (72.4)	60 (78.9)	0.151
	Rectum		92 (27.6)	16 (21.1)	
Histological type	Adenocarcinoma		319 (95.8)	67 (88.2)	0.068
	Mucinous adenocarcinoma		9 (2.7)	5 (6.6)	
	Signet ring cell adenocarcinoma		4 (1.2)	3 (3.9)	
	Carcinoma, NOS		1 (0.3)	1 (1.3)	
Primary tumor	Lymphovascular invasion	Negative	284 (85.3)	54 (71.1)	0.004
			Positive	49 (14.7)	22 (28.9)	
	Perineural invasion	Negative	321 (96.4)	72 (94.7)	0.344
		Positive	12 (3.6)	4 (5.3)	
	Grade^a^	Lower	306 (91.9)	63 (82.9)	0.019
		High	27 (8.1)	13 (17.1)	
Pathologic staging	pT	1	24(7.2)	4(5.3)	0.084
		2	40(12.0)	10(13.2)	
		3	240(72.1)	48(63.2)	
		4	20(8.7)	14(18.4)	
	pN	N0	193 (58.0)	28 (36.8)	0.001
		N+	140 (42.0)	48 (63.2)	
Preoperative serum CEA level (ng/mL)	<10		268 (82.7)	54 (74.0)	0.063
	≥10		56 (17.3)	19 (26.0)	
	Data available		324	73	
Radiologic finding of PALNs	Number	Single	47 (14.1)	7 (9.2)	0.171
		Multiple	286 (85.9)	69 (90.8)	
	Side	Unilateral	98 (29.4%)	27 (35.5)	0.183
		Bilateral	235 (70.6)	49 (64.5)	
Treatment	Best supportive care		204 (61.3)	27 (35.5)	<0.001
	Neoadjuvant chemotherapy		4 (1.2)	3 (3.9)	0.123
	Neoadjuvant CCRT		20 (6.0)	7 (9.2)	0.218
	Postoperative chemotherapy		120 (36.0)	45 (59.2)	<0.001

^a^Lower grade represents well or moderately differentiated histology and high grade represents poorly differentiated histology or mucinous carcinoma.

*PALNs*, *para-aortic lymph nodes; CRC*, *colorectal cancer; NOS*, *not otherwise specified; CEA*, *carcinoembryonic antigen; CCRT*, *concurrent chemoradiotherapy*.

**Fig 3:**
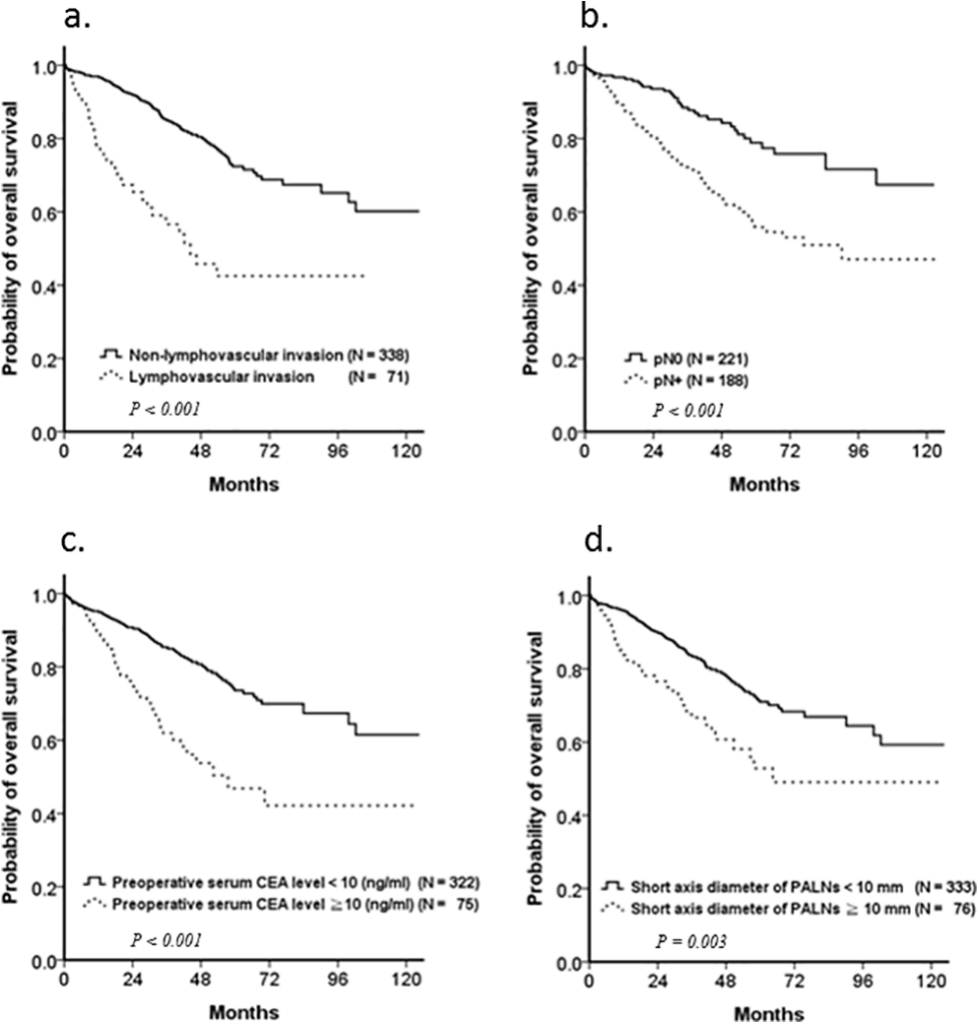
Prognostic factors for overall survival (OS) in patients with visible PALNs. Kaplan–Meier plots revealed that (a) lymphovascular invasion (*P* < 0.001), (b) pN stage (*P* < 0.001), (c) preoperative serum CEA level (*P* < 0.001), and (d) short axis diameter of PALNs (*P* = 0.003) were independent prognostic factor in patients with visible PALNs.

To identify independent prognostic factors, the variables affecting survival were examined by univariate and multivariate Cox proportional hazards regression analyses (Table 4). In the univariate analyses, factors associated with poor survival were pathologic features of the primary tumor (lymphovascular invasion, *P* < 0.001; perineural invasion, *P* = 0.001; higher grade, *P* = 0.033), pathologic staging (pN+, *P* < 0.001), elevated preoperative serum CEA level (*P* ≥ 0.001), and visible PALNs ≥10 mm (*P* = 0.003). Although postoperative chemotherapy was a significant prognostic factor for OS in patients with visible PALNs in univariate analysis (*P* = 0.039), it was insignificant in multivariate analysis (*P* = 0.284). Only lymphovascular invasion (HR = 1.865; 95% CI = 1.112–3.082; *P* = 0.015), pN+ status (HR = 2.099; 95% CI = 1.231–3.578; *P* = 0.006), elevated preoperative serum CEA level (HR = 2.263; 95% CI = 1.470–3.484; *P* < 0.001), and visible PALNs ≥10 mm (HR = 1.638; 95% CI = 1.047–2.563; *P* = 0.031) were independent prognostic factors for OS in multivariate regression analysis (Table 4 and Fig 3).

**Table 4:** Univariate and multivariate Cox regression analysis of prognostic factors for overall survival in patients with visible PALNs.

Characteristics (N = 409)		Univariate	Multivariate	
		*P* value	*P* value	HR (95% CI)
Age (years) ≥ 65		0.067		
Male		0.352		
Rectal cancer		0.814		
Primary tumor	Lymphovascular invasion	<0.001	0.015	1.865 (1.112–3.082)
	Perineural invasion	0.001	0.066	2.151 (0.951–4.862)
	High grade^a^	0.033	0.374	1.293 (0.734–2.277)
Pathologic staging	pT >2	0.259		
	pN +	<0.001	0.006	2.099 (1.231–3.578)
Preoperative serum CEA level ≥10 (ng/mL)		<0.001	<0.001	2.263 (1.470–3.484)
Radiologic finding of PALNs	Short axis ≥10 mm	0.003	0.031	1.638 (1.047–2.563)
	Multiple	0.642		
	Bilateral	0.092		
Treatment	Neoadjuvant chemotherapy	0.891		
	Neoadjuvant CCRT	0.469		
	Postoperative chemotherapy	0.039	0.284	0.759 (0.458–1.258)

^a^High grade represents poorly differentiated histology or mucinous carcinoma.

*PALNs*, *para-aortic lymph nodes; HR*, *hazard ratio; CI*, *confidence interval; CEA*, *carcinoembryonic antigen; CCRT*, *concurrent chemoradiotherapy*.

### The prognostic model for patients with visible PALNs

Having identified independent prognostic factors, we established a prognostic scoring system. Each prognostic factor scored one point, and a prognostic model total score ranging from 0–4 was established. Patients with visible PALNs were divided into three groups: lower-risk (prognostic score of 0–1), intermediate-risk (prognostic score of 2), and high-risk (prognostic score of 3–4). 5-year OS of lower-, intermediate-, and high- risk groups were 78%, 54%, and 25%, respectively (*P* < 0.001).

**Fig 4:**
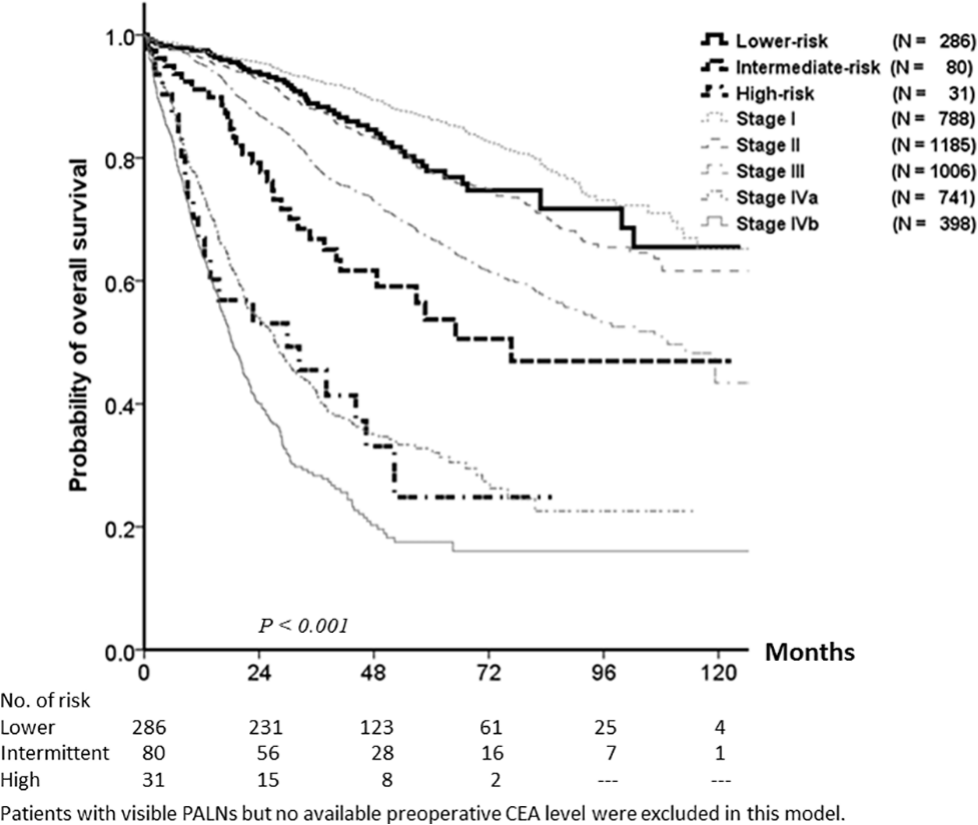
Prognostic model for patients with visible PALNs. The prognostic scoring system scored one point for the presence of each of the following risk factors: lymphovascular invasion, pN+ status, serum carcinoembryonic antigen (CEA) level ≥10 ng/mL, and short axis diameter of PALNs ≥10 mm. According to the total prognostic score, patients with visible PALNs were divided into three groups: lower-risk (prognostic score of 0–1), intermediate-risk (prognostic score of 2), and high-risk (prognostic score of 3–4). 5-year OS of the lower-, intermediate-, and high-risk groups were, 78%, 54%, and 25% respectively (*P* <0.001).

Compared to patients without visible PALNs, the estimated OS of the lower-risk and high-risk groups were the same as the estimated OS of patients with early stage CRC and stage IV disease respectively. The estimated OS of the intermediate-risk group was between stage III and IVa CRC patients (Fig 4).

## Discussion

According to the AJCC staging system and previous studies, the clinical prognostic role and management of visible PALN metastases were equivocal [5, 7], but with the improvement of imaging modalities, these were not rare metastatic patterns in CRC. In our study, visible PALNs were not an independent prognostic factor for OS in patients without distal metastases. To address the prognostic role for these patients, we established a prognostic model to predict the outcome for patients with visible PALNs. Lymphovascular invasion, pN+ status, elevated preoperative serum CEA levels, and visible PALNs >10 mm were identified as independent prognostic factors. After assigning the presence of each prognostic factor one point, patients with visible PALNs were divided into low- (prognostic score of 0–1), intermediate- (prognostic score of 2), and high-risk (prognostic score of 3–4) groups. Only the estimated survival of the intermediate- and high-risk groups was similar to that of patients with stage IV CRC.

Actually, clinicians are uncertain as to whether visible PALNs should be treated as distant metastatic lesions or as regional lymph nodes. The optimal treatment strategies remain inconclusive [4, 10], and there are some issues that lead to undefined treatment strategies. Our findings partially answer these questions.

The cut-off level for the short-axis diameter of clinical PALN metastases has not been well addressed. The detectable sizes of lymph nodes with CT/MRI are dissimilar in different institutions [20, 21]. In our institution, visible PALNs >2 mm would be detectable and mentioned in the image records, but the clinical significance of these visible PALNs should be evaluated. Generally, it is proposed that 10 mm is the acceptable cut-off level for the maximal short-axis diameter of visible PALNs [22]. However, some visible PALNs of less than 10 mm might contain malignant cells, and the sensitivity/specificity of radiologic examination alone for lymph node metastasis is low [23, 24]. Due to the lower sensitivity/specificity of CT/MRI for visible PALNs [22–24], it was difficult to separate benign from malignant nodes with imaging studies; because a low size threshold provides higher sensitivity with low specificity, and a higher size threshold lowers the sensitivity but improves specificity, the evaluation for visible PALNs should depend on other biomarkers. In addition, due to the difficulties of surgical dissection [11], it is hard to prove pathologic metastases of PALNs. To date, a correlation between the size of PALNs and pathological status has not been reported. By survival analysis, our findings showed that 10 mm could be an acceptable cut-off level for PALNs in patients with CRC.

Additionally, the impact of visible PALNs on survival had not been clarified prior to the present study [4, 10]. Although some scattered case reports discuss the prognoses of patients with PALNs, most of them focus on the recurrence and treatment of PALNs [7–9] but not the initial presentation. According to the AJCC staging system [5], PALNs were classified as non-regional lymph nodes, and patients with PALNs were considered clinical stage IV; nevertheless, the characteristics of PALNs were not mentioned in the AJCC staging system. Although regional lymph node size has been used as a strong prognostic factor [25], from past experience, it remains equivocal whether the lymph node size alone could be a good predictor of regional nodal metastases [26, 27]. Concurrent evaluation of biological factors, such as clinicopathologic characteristics, is advised for the prediction of regional nodal metastases [28]. In our study, we point out that although the presence of visible PALNs ≥10 mm is indeed an independent prognostic factor, other biological features such as lymphovascular invasion, pN+ status, and elevated preoperative serum CEA level are also important, and should be considered concurrently.

How to manage visible PALNs is unclear in clinical practice. Previously, the presence of visible PALNs >10mm was an important cut-off level [22]. Visible PALNs <10 mm were considered benign lesions. The treatment for visible PALNs >10mm was inconclusive or controversial, and depended on the discussions of multidisciplinary teams. The prognostic value of visible PALNs needs to be evaluated first. Because the dissection of PALNs is difficult, and the incidence of postoperative complications after extensive lymph node dissection is relatively high, the optimal treatment strategy remains inconclusive [4, 10]. We therefore established a prognostic model to guide the treatment for these patients. According to the prognostic model in our study, patients with lower-risk visible PALNs had similar OS to patients diagnosed with stage I/II CRC, and should be managed as early CRC. The outcome of the intermediate-risk group, is similar to OS of patients diagnosed with CRC between stages III and IV. The treatment is therefore controversial, and more intensive treatments (such as radiotherapy) could be considered. Small cohort studies revealed concurrent chemo-radiotherapy might be useful in treating patients with visible PALNs [7, 29]. However, the development of optimal treatments for intermediate- and high-risk groups requires further study, since there is currently not enough clinical evidence to help guide appropriate treatments for these two groups. In our model,it has been suggested that the following clinicopathologic features, which have also been shown to be predictive factors for nodal metastases, be determined first: lymphovascular invasion, pN+ status, and preoperative CEA levels [30–32]. And we suggest that neo-debulking surgery +/- PALN dissection, followed by involved-field radiation therapy should be considered for patients in intermediate- and high-risk groups with visible PALNs. Patient management determined by AJCC staging system alone might be inadequate.

There were some limitations in this study. The first was the selection bias of visible PALNs; this was a retrospective study, and the limitations of detection of different radiologic machines varied. And example images are shown in S1 Fig. In addition, due to the small number of high-risk group patients, it was difficult to validate the prognostic model [33]. Our findings will require further validation through additional studies. Finally, in our hospital, dissection of PALNs is not a routine technique. The presence of additional risk factors for PALNs did not indicate truly pathologic PALN metastases. The sensitivity and specificity of this prognostic model should help accumulate more pathologic findings in patients with visible PALNs, and detailed studies should be performed to analyze these issues.

In conclusion, lymphovascular involvement, pN+ status, preoperative CEA levels ≥ 10 ng/ml, and the size of the visible PALNs ≥ 10mm were independent prognostic factors for patients with visible PALNs in CRC. These patients should not be routinely classified as AJCC stage IV. A prognostic model could effectively determine the outcome of patients with visible PALNs, and aggressive treatments could be considered for intermediate- and high-risk patients.

## Supporting Information

S1 Fig Images of visible PALNs. (a,b) Images from a 72-year-old man diagnosed as having pT2N0 ascending colon adenocarcinoma with no lymphovascular invasion (LVI) and a preoperative CEA level of 1.1 ng/mL. The patient survived until the last follow-up, and overall survival (OS) was 43.4 months. Contrast enhanced CT images showed: (a) a clip was placed by endoscopy as a marker for tumor localization; there was focal wall thickening near the clip. (b) A low-risk PALN about 5.0 mm in short-axis diameter at the left para-aortic region (white arrow). (c,d) Images from a 31-year-old man diagnosed as having pT4N2 sigmoid colon adenocarcinoma with LVI and a preoperative CEA level of 2264.0 ng/mL. The images showed: (c) circumferential wall thickening of the sigmoid colon with extension through the colonic wall and invasion of the left psoas muscle; (d) a round-shaped high-risk enlarged lymph node, about 20.0 mm in size, was found at the left para-aortic region(black arrow). Left hydronephrosis due to the tumor compressing the left lower third ureter was observed. The patient died, with the overall survival being only 13.8 months.
(TIF)

S1 Table Characteristics of patients with diagnosed CRC at Taipei Veterans General Hospital between January 1, 2001 and December 31, 2011. (DOC)
