# Supplementary figures and images for: The Prognostic Role of Para-Aortic Lymph Nodes in Patients with Colorectal Cancer: Is It Regional or Distant Disease?
Source: PLoS One. 2015 Jun 26;10(6):e0130345. doi: 10.1371/journal.pone.0130345 (PMC4482546; doi:10.1371/journal.pone.0130345)

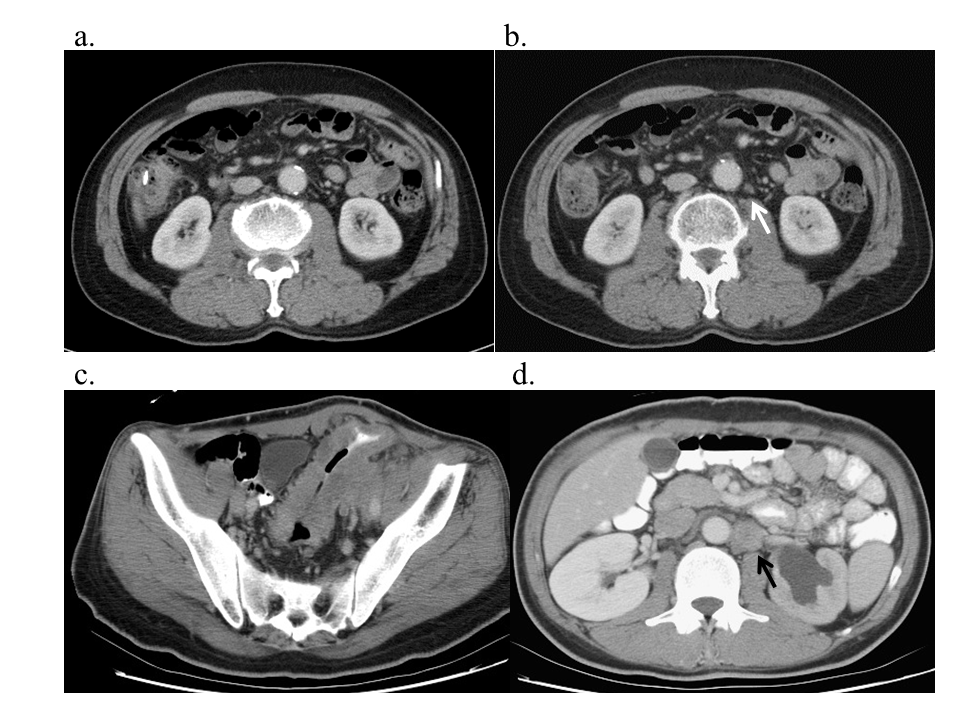

Supplement: S1 Fig — (a,b) Images from a 72-year-old man diagnosed as having pT2N0 ascending colon adenocarcinoma with no lymphovascular invasion (LVI) and a preoperative CEA level of 1.1 ng/mL. The patient survived until the last follow-up, and overall survival (OS) was 43.4 months. Contrast enhanced CT images showed: (a) a clip was placed by endoscopy as a marker for tumor localization; there was focal wall thickening near the clip. (b) A low-risk PALN about 5.0 mm in short-axis diameter at the left para-aortic region (white arrow). (c,d) Images from a 31-year-old man diagnosed as having pT4N2 sigmoid colon adenocarcinoma with LVI and a preoperative CEA level of 2264.0 ng/mL. The images showed: (c) circumferential wall thickening of the sigmoid colon with extension through the colonic wall and invasion of the left psoas muscle; (d) a round-shaped high-risk enlarged lymph node, about 20.0 mm in size, was found at the left para-aortic region(black arrow). Left hydronephrosis due to the tumor compressing the left lower third ureter was observed. The patient died, with the overall survival being only 13.8 months. (TIF) [file pone.0130345.s001.tif]
