# Supplementary material for: The Prognostic Role of Para-Aortic Lymph Nodes in Patients with Colorectal Cancer: Is It Regional or Distant Disease?
Source: PLoS One. 2015 Jun 26;10(6):e0130345. doi: 10.1371/journal.pone.0130345 (PMC4482546; doi:10.1371/journal.pone.0130345)
Supplement: S1 Table — (DOC) [file pone.0130345.s002.doc]

S1 Table. Characteristics of patients with diagnosed CRC at Taipei Veterans General Hospital between January 1, 2001 and December 31, 2011

|  | Stage I | Stage II | Stage III | Stage IVa | Stage IVb | Visible PALNs^a^ |
| --- | --- | --- | --- | --- | --- | --- |
| Characteristics (N = 4527) | N = 788 | N = 1185 | N = 1006 | N = 741 | N = 398 | N = 409 |
| Age (years)  ＜65  ≥ 65 | 310 (39.3%)  478 (60.7%) | 380 (32.1%)  805 (67.9%) | 385 (38.3%)  621 (61.7%) | 336 (45.3%)  405 (54.7%) | 172 (43.2%)  226 (56.8%) | 171 (41.8%)  238 (58.2%) |
| Gender  Male  Female | 484 (61.4%)  304 (38.6%) | 768 (64.8%)  417 (35.2%) | 626 (62.2%)  380 (37.8%) | 486 (65.6%)  255 (34.4%) | 248 (62.3%)  150 (37.7%) | 281 (68.7%)  128 (31.3%) |
| Location  Colon  Rectum | 502 (63.7%)  286 (36.3%) | 932 (78.6%)  253 (21.4%) | 758 (75.3%)  248 (24.7%) | 565 (76.2%)  176 (23.8%) | 330 (82.9%)  68 (17.1%) | 301 (73.6%)  108 (26.4%) |
| Histological type  Adenocarcinoma  Mucinous adenocarcinoma  Signet ring cell adenocarcinoma  Carcinoma, NOS | 775 (98.4%)  8 (1.0%)  2 (0.3%)  3 (0.4%) | 1118(94.3%)  54 (4.6%)  11 (0.9%)  2 (0.2%) | 924 (91.8%)  53 (5.3%)  27 (2.7%)  2 (0.2%) | 688 (92.8%)  41 (5.5%)  9 (1.2%)  3 (0.4%) | 329 (82.7%)  53 (13.3%)  13 (3.3%)  3 (0.8%) | 286 (94.4%)  14 (3.4%)  7 (1.7%)  2 (0.5%) |
| Primary tumor  Lymphovascular invasion  Negative  Positive  Available patients^b^  Perineural invasion  Negative  Positive  Available patients^3^  Grade^c^  Lower  High  Available patients^3^ | 764 (97.0%)  24 (3.0%)  788  784 (99.5%)  4 (0.5%)  788  772 (98.0%)  16 (2.0%)  788 | 1131(95.4%)  54 (4.6%)  1185  1159(97.8%)  26 (2.2%)  1185  1094(92.3%)  91 (7.7%)  1185 | 750 (74.6%)  256 (25.4%)  1006  943 (93.7%)  63 (6.3%)  1006  868 (86.3%)  138 (13.7%)  1006 | 403 (57.5%)  298 (42.5%)  701  615 (87.9%)  85 (12.1%)  700  596 (84.2%)  112 (15.8%)  708 | 160 (48.0%)  173 (52.0%)  333  294 (88.3%)  39 (11.7%)  333  253 (73.3%)  92 (26.7%)  345 | 338 (82.6%)  71 (17.4%)  409  393 (96.1%)  16 (3.9%)  409  369 (90.2%)  40 (9.8%)  409 |
| Preoperative serum CEA level (ng/ml)  ＜ 10  ≥ 10  Available | 738 (97.5%)  19 (2.5%)  757 | 938 (83.3%)  188 (16.7%)  1126 | 748 (77.9%)  212 (22.1%)  960 | 262 (36.3%)  460 (63.7%)  722 | 156 (41.0%)  222 (59.0%)  376 | 322 (81.1%)  75 (18.9%)  397 |
| 5-year overall survival | 86% | 77% | 67% | 33% | 17% | 67% |

^a^Patients with visible PALNs but without distal metastases were recorded in this group.

^b^Due to the performance of only palliative operation for stage IV patients, not all patients had the histologic features of primary tumors.

^c^Lower grade represents well or moderately differentiated histology and high grade represents poorly differentiated histology or mucinous carcinoma.

*PALNs, para-aortic lymph nodes; CRC, colorectal cancer; NOS, not otherwise specified; CEA, carcinoembryonic antigen*
